# Comparative Efficacy and Safety of Topical Clobetasol Propionate 0.05% Ointment Versus Topical Methotrexate 1% Gel in Limited Plaque Psoriasis: A Prospective Open-Label Study

**DOI:** 10.7759/cureus.105980

**Published:** 2026-03-27

**Authors:** Radhika Thakur, Dinesh P Asati, Kapila Verma, Shreya Sharma, Vishesh Budhwani

**Affiliations:** 1 Dermatology, LN Medical College and Research Center, Bhopal, IND

**Keywords:** comparative study, limited plaque psoriasis, pasi, topical corticosteroid, topical methotrexate

## Abstract

Background

Psoriasis is a chronic, inflammatory, immune-mediated skin disorder characterized by abnormal keratinocyte proliferation and systemic inflammation. Limited plaque psoriasis, defined as involving less than a specified portion of the body surface area (BSA), presents as localized, erythematous, and scaly plaques. Though milder in extent, it significantly impairs quality of life due to physical discomfort and psychological distress. Topical therapies remain the primary treatment approach for this subtype. Potent topical corticosteroids, such as clobetasol propionate 0.05% ointment, offer rapid relief but pose long-term risks, like skin atrophy. In contrast, topical methotrexate 1% gel is emerging as a safer, sustained alternative.

Objective

The primary objective of this study was to compare the efficacy and safety of topical methotrexate 1% gel versus clobetasol propionate 0.05% ointment in patients with limited plaque psoriasis. Secondary objectives included the assessment of disease severity scores and adverse effects.

Methods

This prospective comparative, open-label study involved a per-protocol cohort of 70 patients. Patients were divided into two groups: clobetasol, 35 (50%) patients, and methotrexate, 35 (50%) patients, and were followed for 12 weeks.

Results

Clobetasol demonstrated faster initial clinical improvement by Week 4, but methotrexate yielded significantly better sustained results. By Week 12, 13 (37.1%) patients in the methotrexate group achieved Psoriasis Area and Severity Index (PASI)-75, compared to five (14.3%) in the clobetasol group (p = 0.026). Endline Physician's Global Assessment (PGA) scores showed seven (20%) methotrexate patients achieved clear skin, and 19 (54.3%) were almost clear, contrasting favorably against clobetasol (two (5.71%) clear; nine (25.7%) almost clear). Methotrexate significantly improved Beer Sheva Psoriasis Severity Score (BSPSS) values without serious adverse events. The clobetasol group reported atrophy in eight (22.9%) patients and tachyphylaxis in one (2.9%) patient at Week 10.

Conclusion

Topical methotrexate 1% gel is significantly superior to clobetasol propionate for sustained treatment. It provides high clinical efficacy and avoids steroid-associated adverse effects, making it a highly recommended, safer, long-term option for limited plaque psoriasis.

## Introduction

Psoriasis is a chronic, inflammatory, immune-mediated skin disorder with a significant worldwide prevalence, estimated to affect approximately 2%-3% of the global population [1,2]. The prevalence in India specifically ranges from 0.44% to 2.8%, making it a considerable public health concern [3,4]. The pathogenesis involves a complex interplay of genetic predisposition, environmental triggers, and immune dysregulation, leading to abnormal keratinocyte proliferation and altered immune responses [5].

Clinically, limited plaque psoriasis is a milder subtype involving less than 10% of the body surface area (BSA) and presenting as localized, erythematous, and scaly plaques. Despite the limited BSA, the condition significantly impacts patients' quality of life due to physical discomfort, pruritus, and psychological distress [6,7]. Furthermore, psoriasis is known to be associated with systemic comorbidities, such as metabolic syndrome, cardiovascular disease, and psoriatic arthritis [8,9]. While these comorbidities are most heavily associated with moderate-to-severe disease, they are also recognized as prevalent risks in patients with limited plaque psoriasis, necessitating comprehensive clinical management.

Topical therapies serve as the cornerstone of treatment for this subtype, offering localized action and a lower risk of systemic side effects. Potent topical corticosteroids, such as clobetasol propionate 0.05% ointment, are widely used for rapid anti-inflammatory and antiproliferative effects [10]. However, prolonged use is associated with local side effects, like skin atrophy, telangiectasia, striae, and tachyphylaxis, which necessitate short or intermittent application schedules [11-14]. In contrast, topical methotrexate 1% gel is emerging as a safer, long-term alternative for mild-to-moderate psoriasis [15,16]. It functions by inhibiting DNA synthesis and keratinocyte hyperproliferation, mitigating the severe systemic risks associated with oral methotrexate [17,18].

While super-potent topical corticosteroids enjoy widespread FDA and EMA approval for plaque psoriasis, topical methotrexate 1% gel is largely utilized as an off-label alternative, or under specific regional regulatory approvals. Where available, it represents a highly cost-effective alternative to newer non-steroidal agents and biologics. Its utility also extends to other localized inflammatory skin disorders, with local side effects generally limited to mild, transient irritation. Despite its potential, there is a distinct research gap: there are few comparative studies, limited long-term safety data, and a notable lack of studies evaluating its efficacy using the comprehensive Beer Sheva Psoriasis Severity Score (BSPSS) tool. Therefore, the primary objective of this study was to compare the efficacy and safety of topical methotrexate 1% gel versus clobetasol propionate 0.05% ointment in patients with limited plaque psoriasis.

## Materials and methods

Study design and setting

This study was designed as a single-center, hospital-based, prospective comparative open-label study conducted in the Department of Dermatology at LN Medical College and Research Center, Bhopal, India. It was carried out over 18 months.

Sample size calculation

To ensure adequate statistical power, the sample size was calculated prior to enrollment. To achieve a power of 80% and a confidence level of 95%, based on expected differences in Psoriasis Area and Severity Index (PASI)-75 achievement rates between interventions, a final per-protocol sample size of 70 patients was required. Initially, 78 patients were enrolled to account for potential dropouts; eight patients were excluded from the final evaluation due to being lost to follow-up or protocol noncompliance.

Study population and group allocation

Participants were recruited using a non-probability convenience sampling technique. The final 70 participants were divided into two equal groups of 35 patients each. Group allocation was determined after mutual discussion between the investigator and the patient regarding treatment suitability and preference. This discussion prominently factored in patient treatment history, as those with extensive prior topical corticosteroid use frequently exhibited “steroid fatigue,” which drove a strong preference for the non-steroidal methotrexate arm.

Regimen and interventions

Group 1 received topical clobetasol propionate 0.05% ointment, while Group 2 received topical methotrexate 1% gel. To ensure standardized dosing and methodological reproducibility, the application quantity of both agents was guided by the Fingertip Unit (FTU) method, in which one FTU (approx. 0.5 g) was designated to cover roughly 2% of the BSA. The prescribed FTUs were individually calculated based on the patient's baseline BSA and applied twice daily. To prevent confounding, no concomitant moisturizers or adjunct active therapies were permitted during the 12-week study period.

Inclusion and exclusion criteria

Patients aged 18-60 with stable limited plaque psoriasis (<10% BSA) were included. Patients were excluded if they presented with guttate, pustular, erythrodermic, or inverse/flexural psoriasis. Inverse psoriasis was strictly excluded because natural occlusion in flexural areas drastically alters the absorption rate and side-effect profile of super-potent corticosteroids, introducing severe confounding risks. Furthermore, to prevent residual pharmacological effects, any patients who had recently used topical or systemic anti-psoriatic therapies were required to undergo a mandatory, two-week washout period prior to baseline assessment.

Outcome measures and assessments

Treatment efficacy was assessed using the PASI [19], the Physician's Global Assessment (PGA) [20], and the BSPSS [21] at baseline, Week 4, Week 8, and Week 12. To minimize inter-observer variability, all clinical evaluations were strictly standardized and performed by the same investigator. Furthermore, patient adherence to the twice-daily treatment regimen was rigorously monitored during these scheduled outpatient evaluations to ensure compliance over the 12-week period.

Ethical considerations and statistical analysis

Ethical approval was obtained (approval No: LNMC&RC/Dean/2023/Ethics/214). Data were analyzed using Stata version 17.0 (StataCorp LLC, College Station, TX, USA). Continuous variables were evaluated using independent Student's t-tests and paired t-tests. The chi-square test was used to compare categorical variables, and repeated-measures analysis of variance (ANOVA) was used to evaluate longitudinal changes. A p-value of <0.05 was considered statistically significant.

## Results

The 70 enrolled patients were well-matched at baseline. Most participants were 21 to 40 years of age, with the back and extensor aspects of the lower limbs being the most common sites of lesions.

Efficacy outcomes and disease duration

**Figure 1:**
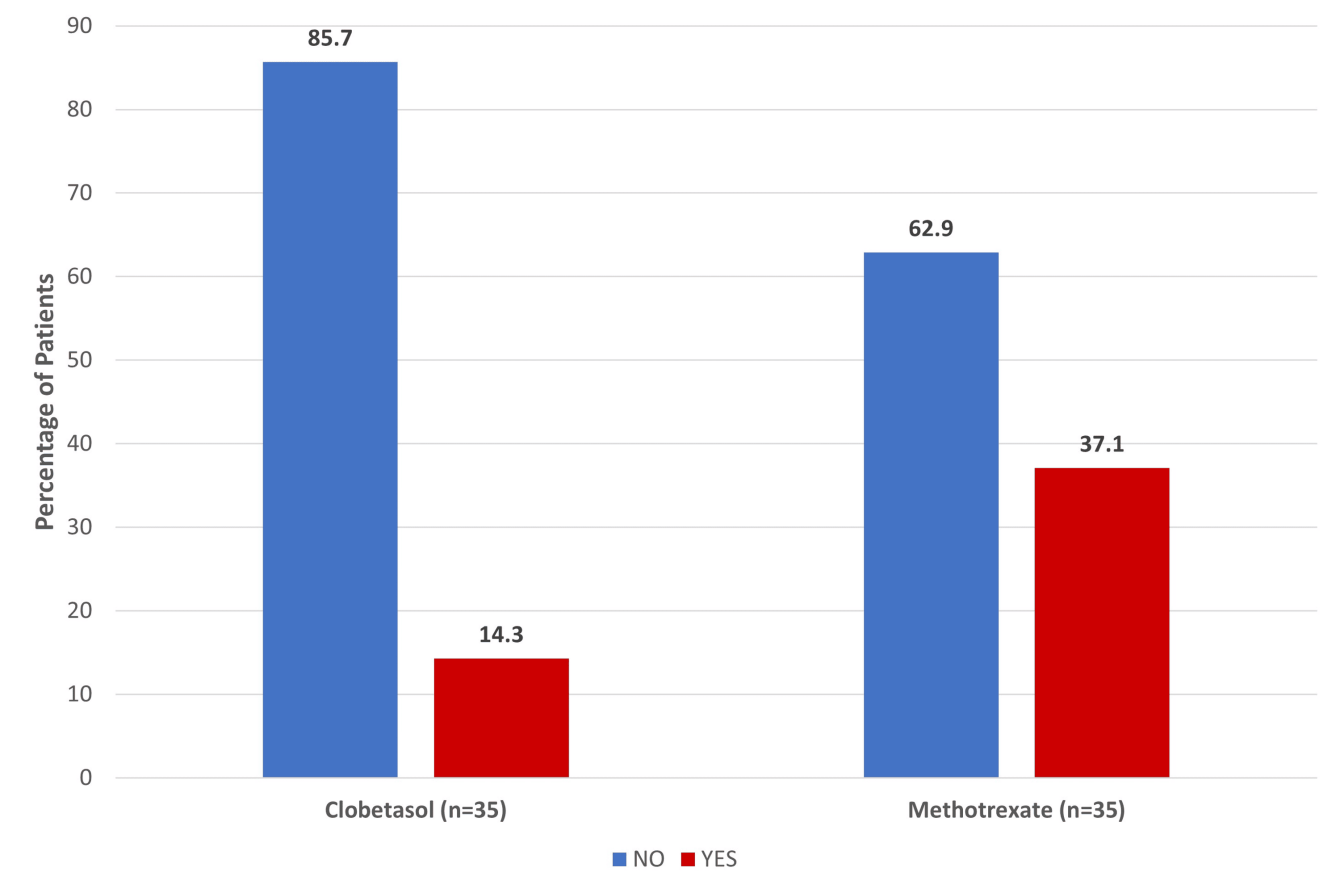
Percentage of Patients Achieving PASI-75 at Week 12 PASI: Psoriasis Area and Severity Index

When factoring in disease duration, stratification revealed that, while chronic, long-standing plaques initially resisted treatment, the methotrexate group maintained a steady clearance rate regardless of disease chronicity. In terms of treatment speed, clobetasol demonstrated faster initial results, showing noticeable PASI reductions by Week 4. However, methotrexate provided far superior sustained results over the 12-week course. Comparative analysis revealed that the methotrexate group showed a statistically significant reduction in mean PASI scores at Week 8 and Week 12 compared with the clobetasol group (Tables 1-2). At Week 12, a higher percentage of participants in the methotrexate group achieved PASI-75, totaling 13 (37.1%) patients, compared with five (14.3%) patients in the clobetasol group (Figure 1).

**Table 1:** Comparative Analysis of Mean PASI Scores Over 12 Weeks PASI: Psoriasis Area and Severity Index

Time Point	Clobetasol (n = 35), Mean ± SD	Methotrexate (n = 35), Mean ± SD	t-value	p-value
Baseline	3.85 ± 0.78	3.98 ± 0.97	0.61	0.5457
Week 4	3.53 ± 0.79	3.57 ± 0.96	0.18	0.8556
Week 8	3.13 ± 0.83	2.50 ± 1.23	2.93	0.0049
Week 12	2.15 ± 1.20	1.51 ± 1.36	2.29	0.026

**Table 2:** Repeated Measures ANOVA (Within-Group Analysis) ANOVA: Analysis of Variance

Group	F-value	p-value
Clobetasol	24.86	<0.001
Methotrexate	38.41	<0.001

Endline PGA scores were also significantly better in the methotrexate group (p < 0.05). A substantial majority of participants in the methotrexate group were rated as clear (seven (20%) patients) or almost clear (19 (54.3%) patients). In contrast, the clobetasol group showed fewer patients in these categories, with only two (5.71%) patients rated as clear and nine (25.7%) patients as almost clear. The remaining participants in the clobetasol group were rated as mild (12 (34.3%) patients), moderate (10 (28.6%) patients), or severe (two (5.71%) patients). In the methotrexate group, only eight (22.9%) patients were rated as mild and one (2.86%) patient as moderate, with no patients in the severe category (Tables 3-4).

**Table 3:** Distribution of Endline Physician's Global Assessment (PGA) Scores at Week 12

Endline PGA Score	Clobetasol (n = 35) n (%)	Methotrexate (n = 35) n (%)
Clear	2 (5.71%)	7 (20%)
Almost Clear	9 (25.7%)	19 (54.3%)
Mild	12 (34.3%)	8 (22.9%)
Moderate	10 (28.6%)	1 (2.86%)
Severe	2 (5.71%)	0 (0%)

**Table 4:** Chi-Square Test

χ² value	df	p-value
16.51	4	0.002

**Figure 2:**
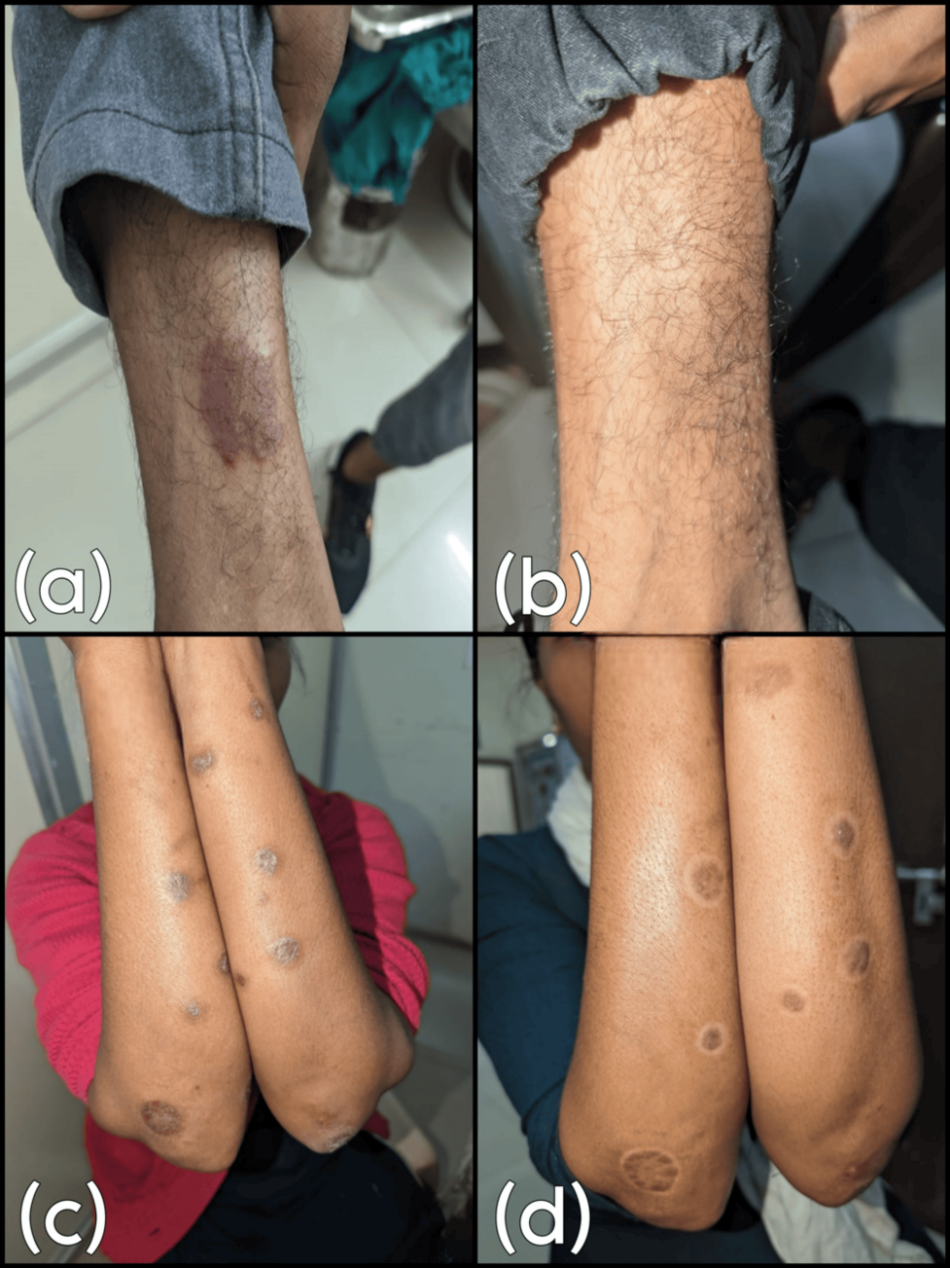
Comparative Clinical Response of Limited Plaque Psoriasis to Topical Therapies Over 12 Weeks Panels (a) and (b) display the target lesion of a representative patient treated with topical methotrexate 1% gel at baseline (a) and Week 12 (b), demonstrating significant plaque resolution and resulting in a “clear” or “almost clear” Physician’s Global Assessment (PGA) score. Panels (c) and (d) display the target lesion of a representative patient treated with clobetasol propionate 0.05% ointment at baseline (c) and Week 12 (d), showing less complete resolution, corresponding to a “moderate” or “mild” PGA score at the study endpoint.

The superior clinical clearance achieved by the methotrexate group, compared to the clobetasol group, over the 12-week study period is visually represented in the composite clinical response image (Figure 2).

Quality of severity improvement and safety

The methotrexate group showed greater improvement in overall psoriasis severity at Week 12, as indicated by lower BSPSS values, compared to the clobetasol group (Tables 5-6). 

**Table 5:** Comparative Analysis of Mean BSPSS Scores Over 12 Weeks BSPSS: Beer Sheva Psoriasis Severity Score

Time Point	Clobetasol (n = 35) Mean ± SD	Methotrexate (n = 35) Mean ± SD	t-value	p-value
Baseline	8.72 ± 1.35	8.89 ± 1.42	0.49	0.6214
Week 4	7.95 ± 1.40	7.78 ± 1.51	0.47	0.6412
Week 8	6.82 ± 1.56	5.74 ± 1.68	2.83	0.0061
Week 12	5.21 ± 1.73	3.94 ± 1.88	2.44	0.0183

**Table 6:** Repeated Measures ANOVA (Within-Group BSPSS Change) ANOVA: Analysis of Variance; BSPSS: Beer Sheva Psoriasis Severity Score

Group	F-value	p-value
Clobetasol	27.32	<0.001
Methotrexate	41.75	<0.001

Mean BSPSS scores reduced significantly over time in both treatment groups. At baseline, the mean BSPSS score was 8.72 ± 1.35 in the clobetasol group and 8.89 ± 1.42 in the methotrexate group (p = 0.6214), indicating comparable disease severity.

At Week 4, both groups showed a mild reduction (7.95 ± 1.40 vs. 7.78 ± 1.51; p = 0.6412). However, by Week 8, the methotrexate group demonstrated a significantly greater reduction (5.74 ± 1.68) compared to the clobetasol group (6.82 ± 1.56), with a p-value of 0.0061.

By Week 12, the mean BSPSS score further reduced to 3.94 ± 1.88 in the methotrexate group, compared to 5.21 ± 1.73 in the clobetasol group (p = 0.0183), confirming superior overall severity reduction with topical methotrexate.

Regarding safety, topical methotrexate 1% gel was well tolerated. The most common side effects reported were mild and local: pruritus in five (14.3%) patients, irritation in four (11.4%) patients, and a burning sensation in two (5.71%) patients. No serious or systemic adverse events were observed.

**Figure 3:**
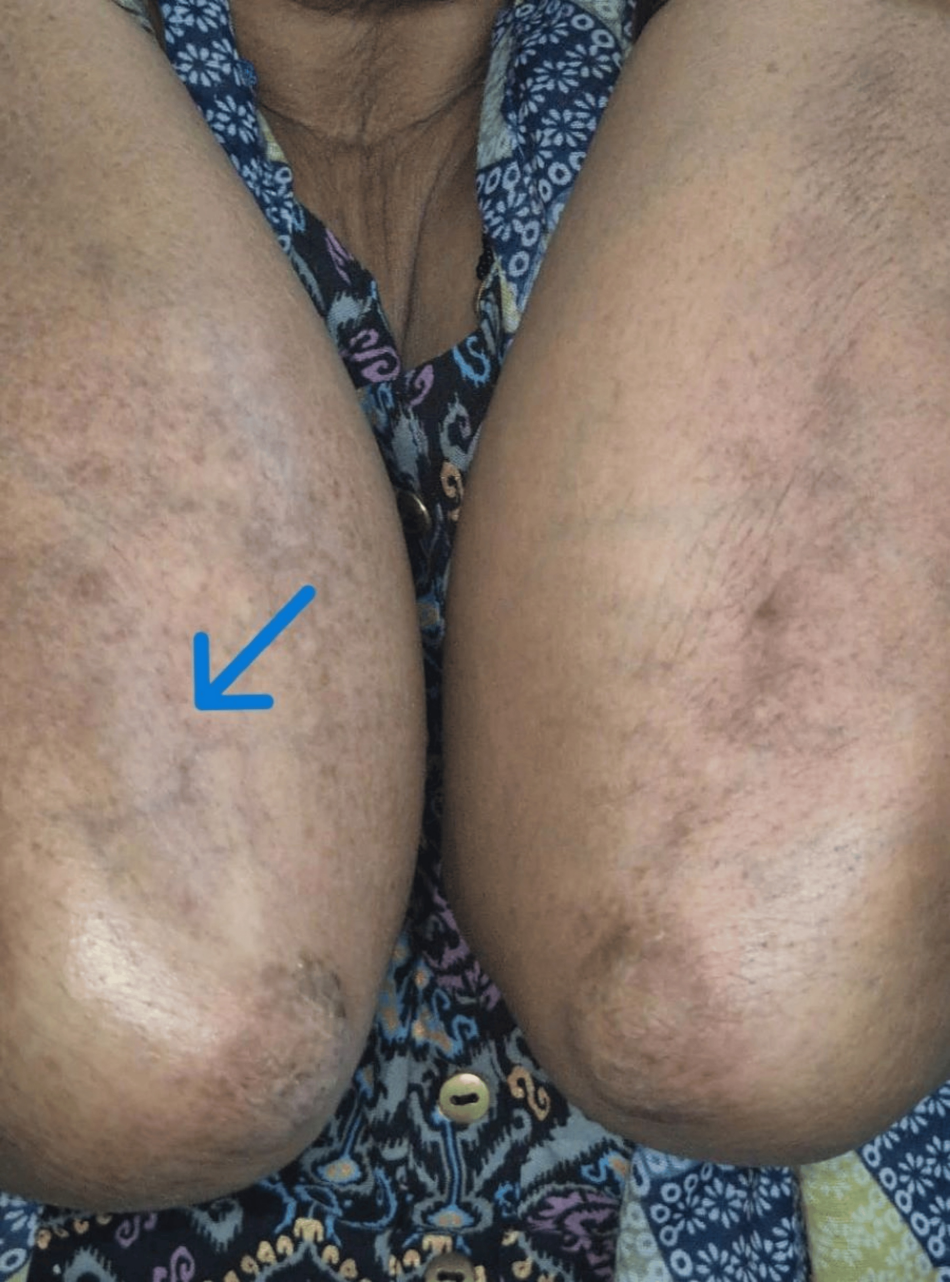
Local Adverse Effect of Potent Topical Corticosteroid Clinical image showing signs of skin atrophy (arrow), following treatment with clobetasol propionate 0.05% ointment.

In contrast, the clobetasol group reported significant steroid-related local side effects. These included skin atrophy in eight (22.9%) patients, striae in five (14.3%) patients, and contact dermatitis in four (11.6%) patients. Notably, one (2.9%) patient in the clobetasol group developed marked tachyphylaxis at Week 10 of treatment, a rare finding for limited BSA application within this specific timeframe (Table 7 and Figure 3).

**Table 7:** Adverse Event Profile by Treatment Group

Adverse Event	Clobetasol (n = 35) n (%)	Methotrexate (n = 35) n (%)	χ² value	p-value
Skin Atrophy	8 (22.9%)	0 (0%)	8.89	0.003
Striae	5 (14.3%)	0 (0%)	5.38	0.02
Telangiectasia	3 (8.6%)	0 (0%)	3.23	0.072
Folliculitis	2 (5.8%)	0 (0%)	2.06	0.151
Acneiform Eruptions	3 (8.6%)	0 (0%)	3.23	0.072
Rosacea	3 (8.6%)	0 (0%)	3.23	0.072
Contact Dermatitis	4 (11.6%)	0 (0%)	4.38	0.036
Tachyphylaxis	1 (2.9%)	0 (0%)	1.01	0.315
Pruritus	0 (0%)	5 (14.3%)	5.38	0.02
Irritation	0 (0%)	4 (11.4%)	4.38	0.036
Burning Sensation	0 (0%)	2 (5.7%)	2.06	0.151

## Discussion

This study establishes the superior comparative efficacy and safety profile of topical methotrexate 1% gel over clobetasol propionate for the sustained treatment of limited plaque psoriasis.

Mechanism and efficacy comparisons

While potent corticosteroids like clobetasol offer fast initial results, their sustainability is strictly limited by side effects. In this study, the clobetasol group experienced significant local complications, including skin atrophy in eight (22.9%) patients. The superior, sustained improvement seen with topical methotrexate is largely due to its mechanism of inhibiting keratinocyte hyperproliferation at the cellular level, providing deeper disease modification without the risks of systemic absorption or localized thinning. This was reflected in the significantly better PASI-75 achievement rates and BSPSS score reductions. Although PASI is widely used, it has limitations in mild psoriasis due to limited sensitivity at low BSA involvement [22]. Therefore, composite severity tools, like the BSPSS, provided a more comprehensive and accurate assessment of disease characteristics in this limited plaque cohort [23].

Clinical implications and steroid fatigue

In real-world clinical practice, patient preference is heavily influenced by prior treatment experiences. Notably, “steroid fatigue” or “steroid phobia” in patients with chronic disease often drove the preference for the non-steroidal methotrexate gel during group allocation. Accommodating this preference was critical for compliance. By Week 12, methotrexate’s local side effects were limited to temporary pruritus in five (14.3%) patients and irritation in four (11.4%) patients. This contrasts sharply with the potentially irreversible side effects seen in the clobetasol cohort.

Cost-effectiveness and long-term use

Given the chronic, relapsing nature of psoriasis, long-term management strategies must balance safety with accessibility. Topical methotrexate represents a highly cost-effective treatment modality when compared to newer non-steroidal topical agents and systemic biologics, securing its place as a viable long-term maintenance option. Despite these advantages, the use of topical methotrexate remains limited in the United States and Europe. This geographical disparity is primarily due to a lack of widespread FDA or EMA commercial approval for pre-formulated topical methotrexate products, which often restricts its availability to specialized compounding pharmacies. In contrast, in regions where commercial gel formulations have obtained regional regulatory approval, they serve as a highly accessible and practical therapeutic option.

Strengths and limitations

The strengths of this study lie in its prospective comparative nature, the real-world clinical applicability of factoring in patient treatment history, and the robust use of multiple severity and quality-of-life scoring systems (PASI, PGA, and BSPSS). However, several limitations must be acknowledged. Group allocation was based on mutual discussion and patient preference rather than strict formal randomization, which introduces an inherent selection bias. Furthermore, the study was an open-label design, utilized convenience sampling, was conducted at a single center, and was limited to a 12-week follow-up period.

Future research directions

To definitively confirm these findings, future research should consider conducting intra-patient "split studies" (right-left comparative designs) to elegantly eliminate inter-individual biological variations, alongside larger, multicenter, double-blind, randomized controlled trials.

## Conclusions

Topical methotrexate 1% gel is significantly superior to clobetasol propionate 0.05% ointment for the sustained treatment of limited plaque psoriasis. While clobetasol provides rapid short-term relief, methotrexate achieves higher PASI-75 clearance rates and significantly improves overall disease severity over a 12-week course. Crucially, methotrexate avoided the irreversible steroid-associated complications observed in the corticosteroid arm, making it a highly efficacious, cost-effective, and safe clinical recommendation for long-term management.
